# Relationships of Non-coding RNA with diabetes and depression

**DOI:** 10.1038/s41598-019-47077-9

**Published:** 2019-07-24

**Authors:** Tian An, Jing Zhang, Yue Ma, Juan Lian, Yan-Xiang Wu, Bo-Han Lv, Meng-Hua Ma, Jun-Hua Meng, Yun-Tao Zhou, Zhi-Yong Zhang, Qing Liu, Si-Hua Gao, Guang-Jian Jiang

**Affiliations:** 1 Traditional Chinese Medicine School, Beijing University of Chinese Medicine, Beijing, 100029 China; 2 Department of Endocrinology, Tangshan Workers Hospital, Tangshan, 063000 China; 3 Beijing Medicine and Food Co., Ltd., Beijing, 100029 China

**Keywords:** Molecular medicine, Diabetes complications, Long non-coding RNAs

## Abstract

In order to study the molecular differences between type 2 diabetes mellitus (T2DM) and T2DM with depression (DD), we aimed to screen the differential expression of lncRNA, mRNA, and circRNA in the blood of patients with T2DM and DD. Based on the self-rating depression scale (SDS), patient health questionnaire 9 (PHQ9), blood glucose and HbA1c, we divided the patients into T2DM and DD group. Peripheral blood was collected from the two groups of patients to perform lncRNA, mRNA, and circRNA expression profiling and screening DD-related specific molecules. Subsequently, bioinformatics analysis was performed to investigate the functions of differentially expressed genes (DEgenes). Finally, RT-PCR and lncRNA-mRNA regulatory network was performed to verify the expressions of lncRNAs and mRNAs related to the occurrence and development of DD. 28 lncRNAs, 107 circRNAs, and 89 mRNAs were identified in DD differential expression profiles. GO and pathway analysis found that 20 biological process (BP) related entities and 20 pathways associated with DD. The analysis shows that the genes that are differentially expressed in the DD group involved in the development of the neuropsychiatric system, immunity, and inflammation. Then, we screening for the important DElncRNA and mRNA associated with DD were verified by RT-PCR experiments and the results of RT-PCR were consistent with the sequencing results. LncRNA, circRNA, and mRNA differential expression profiles exist in DD patients compared with T2DM. The lncRNA-mRNA regulatory network analysis confirmed the crosslinking and complex regulation patterns of lncRNA and mRNA expression and verified the authenticity of the regulatory network.

## Introduction

Type 2 diabetes mellitus (T2DM) and depression have become chronic psychosomatic diseases that pose the most serious threat to human health following cancer, cardiovascular and cerebrovascular diseases and AIDS. One out of every four patients with T2DM is accompanied by significant depressive symptoms^1^. The effects of T2DM with depression (DD) are extremely high and have a higher risk of death than patients with T2DM or depression alone^2^. Depression increases the development of T2DM and the subsequent risk of complications such as hyperglycemia, insulin resistance, microvascular and macrovascular; in contrast, T2DM also increases the risk of depression in the patient and may lead to more severe depression^3,4^. This association reflects a common etiology consisting of complex bidirectional interactions between multiple variables, including autonomic disorders, neurohormonal disorders, weight gain, inflammation, and hippocampal structural changes^5^.

However, the specific molecular mechanisms of the correlation between DD and T2DM are remain unclear. Long non-coding RNAs (lncRNAs) are a group of RNAs between 200 nt and 100 kb in length and have limited protein-coding potential, functioning as regulatory genes at the epigenetic, transcriptional and post-transcriptional levels^6,7^. Recent studies have shown that lncRNAs can participate in a variety of important biological control processes and the development of a variety of diseases, including a variety of neuropsychiatric and metabolic diseases including diabetes and depression^8–10^. CircRNA, a type of closed circular RNA molecule, can play a competitive endogenous RNA (ceRNA) role by regulating transcription and blocking miRNAs’ inhibition of their target genes^11^. CircRNA is involved in the development of many diseases, and the differential expression of circRNAs plays an important role in the disease development^12^. Therefore, understanding the role of lncRNAs and circRNAs in diseases and their mechanisms is an important topic in current research. Therefore, we studied the nature of DD from LncRNA, mRNA and mRNA, and explored the pathological mechanism of DD from the transcriptomic level.

## Results

### Clinical characteristics of the participants

This study enrolled 5 T2DM patients and 5 DD patients. Baseline comparisons were made between this 10 subjects, including gender, age, self-rating depression scale (SDS), and patient health questionnaire 9 (PHQ9) scores (Table 1 and Supplementary Table 1). There were no significant differences between the groups in terms of age. The mean SDS and PHQ-9 were significantly different between the two groups. The average of SDS total coarse points and standard score of DD patients were 45.6 and 56.8, and the average of PHQ-9 was 9.2.

**Table 1:** Clinical characteristics of the participants.

Characteristics	T2DM	DD
Male/Female	1/4	1/4
Age(year)	59.80 ± 11.07	55.80 ± 5.49
SDS Total coarse points	31.60 ± 4.33	45.60 ± 3.78*
SDS Standard score	39.20 ± 5.44	56.80 ± 4.60*
PHQ-9 score	2.80 ± 1.30	9.20 ± 5.16*

n = 5, values are presented as Mean ± SD. Significant differences by *p < 0.05

T2DM, type 2 diabetes mellitus; DD, T2DM with depression; SDS, self-rating depression scale; PHQ9, patient health questionnaire 9.

### Differentially expressed mRNA (DEmRNA) between T2DM and DD

**Figure 1:**
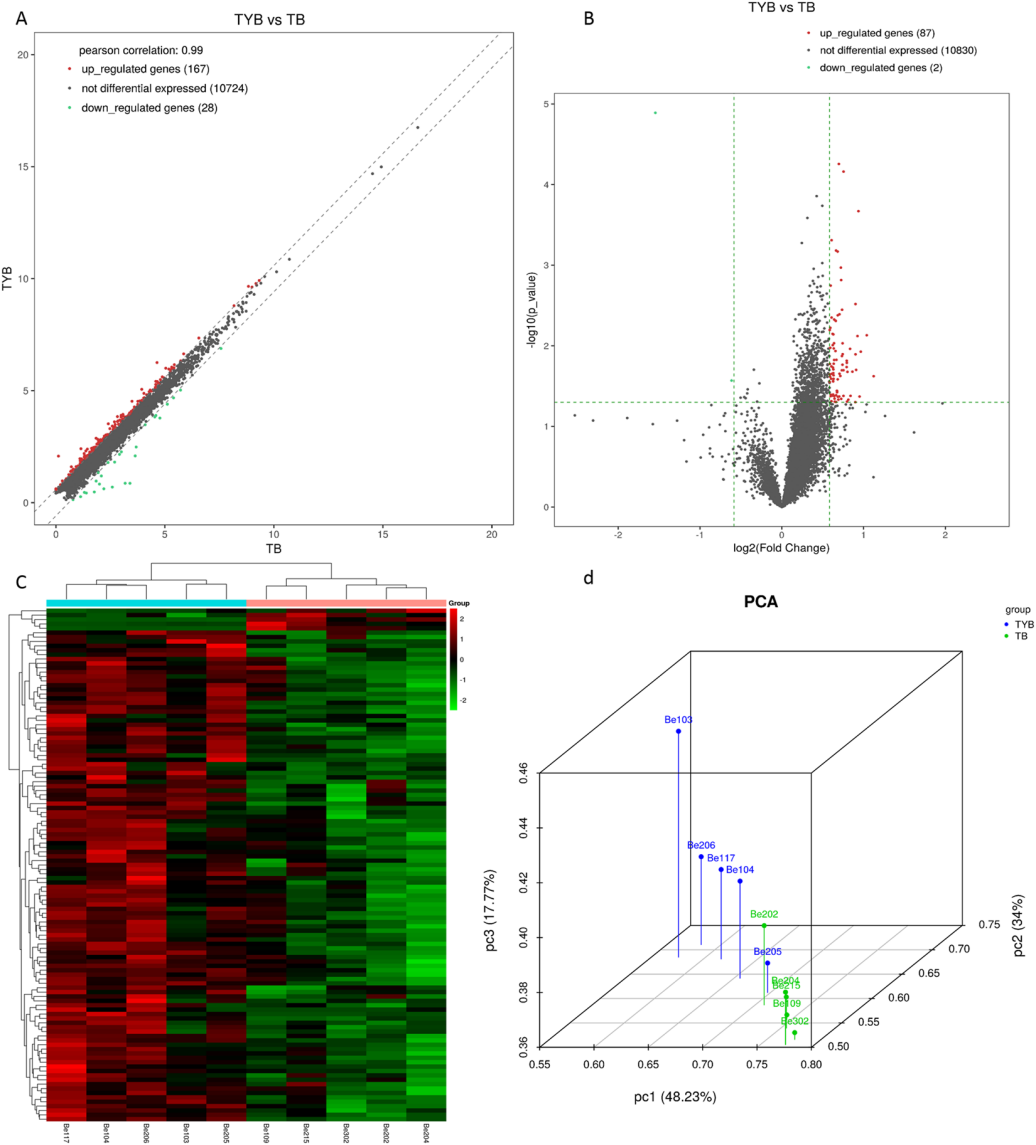
Plots of mRNA. Scatter plot (**A**), the x-axis and y-axis represent the mean FPKM values (log2 transformation) of each group of sample genes. The two oblique lines divide the upper and lower genes (1.5 fold difference) and the unmodified genes. Volcano-Plot (**B**), the x-axis represents the log2Fold_Change value and the y-axis represents -log10p_value. The vertical two green lines are up (right) and down (left), and the green parallel lines correspond to p-value. Hierarchical clustering (**C**) was performed using FPKM values of significant expression genes obtained by comparison between groups, each row representing one gene and each column representing one sample. The red dots represent up-regulated DEgenes, the green dots represent down-regulated DEgenes, and the gray dots represent indistinguishable genes. PCA analysis (**D**), each color represents each grouping, and each axis represents a principal component.

**Figure 2:**
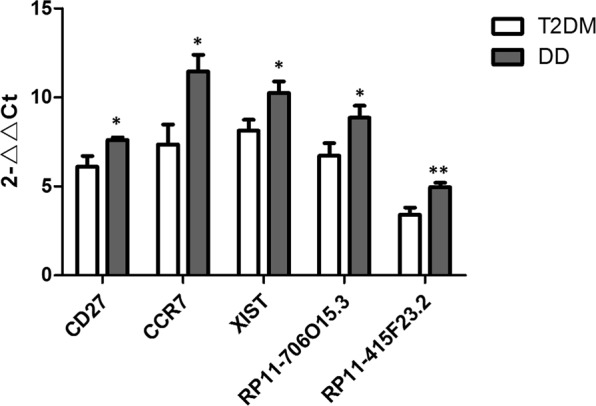
Validation of sequencing data by qRT-PCR. Comparison of DEmRNAs (CD27 and CCR7) and DElncRNAs (XIST, RP11-706O15.3, RP11-415F23.2) qRT-PCR data between groups. *P < 0.05, **P < 0.01 versus T2DM group, N = 10.

Compared with the T2DM group, we found a total of 87 up-regulated and 2 down-regulated mRNAs in the DD group (Fold Change > 1.5, P < 0.05, Supplementary data 1). The up-regulated DEmRNA-OR2W3 had the highest difference (Fold Change = 2.179), and the Fold Change of the two down-regulated mRNAs (UBC and ORMDL3) were 0.653 and 0.343, respectively (Table 2). Among them, the up-regulated expression of CD27 in the mRNA correlates with depression^13^. Therefore, CD27 can be used as an important molecular target for the study of DD. Cluster analysis of DEmRNA showed that these DEmRNAs clearly divided the samples into T2DM and DD groups; in addition, volcano and scatter plots also visually displayed DEmRNA between the two groups (Fig. 1). The expression levels of CD27 and CCR7 selected, as determined by quantitative real-time PCR, were consistent with the sequencing results (Fig. 2), verifying the accuracy and reliability of the sequencing data.

**Table 2:** Top 10 up-regulated mRNAs and down-regulated mRNAs.

Track id	Gene Name	Fold Change	P	q
**Up-regulated**				
ENSG00000238243.3_1	OR2W3	2.179	0.024	0.610
ENSG00000126353.3_2	CCR7	2.054	0.007	0.610
ENSG00000198034.10_1	RPS4X	1.953	0.012	0.610
ENSG00000254996.5_2	ANKHD1-EIF4EBP3	1.933	0.043	0.610
ENSG00000184613.10_2	NELL2	1.915	0.000	0.448
ENSG00000139193.3_2	CD27	1.899	0.008	0.610
ENSG00000101224.17_1	CDC25B	1.878	0.020	0.610
ENSG00000197153.4_2	HIST1H3J	1.868	0.013	0.610
ENSG00000103064.13_2	SLC7A6	1.868	0.003	0.610
ENSG00000256235.1_1	SMIM3	1.864	0.050	0.610
**Down-regulated**				
ENSG00000150991.14_2	UBC	0.653	0.027	0.610
ENSG00000172057.9_2	ORMDL3	0.343	0.000	0.204

### Differentially expressed circRNAs (DEcircRNAs) between T2DM and DD

**Figure 3:**
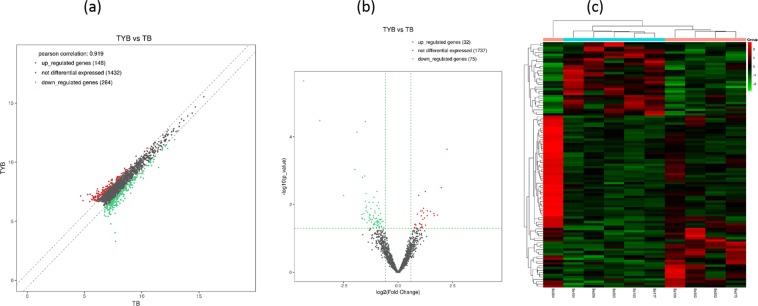
Plots of circRNA. Scatter plot (**a**), volcano-Plot (**b**) and hierarchical clustering (**c**). X-axis and y-axis represent CPM mean values (log2 transitions) of two groups. The red point in the plot represents the DEgenes with statistical significance.

Compared with the T2DM group, 75 down-regulated and 32 up-regulated circRNAs were detected in the DD group (Fig. 3, Supplementary data 2). As shown in Table 3, we listed the top 10 circRNAs with up-regulation and down-regulation, respectively, based on log2 (Fold Change). Compared with the T2DM group, circRNA-TFRC and TXLNG2P were the most significantly up-regulated and down-regulated expressions in the DD group, respectively. Therefore, we speculate that these DEcircRNAs may affect depression in T2DM patients through many molecular mechanisms.

**Table 3:** Top 10 up-regulated and down-regulated circRNAs.

circRNA ID	Gene Name	Length	log2FC	Fold Change	P value
**Up-regulated**					
chr3:195781950-195782172:-	TFRC	222	2.244	4.738	0.000
hsa_circ_0059684	ZNF337-AS1	253	1.983	3.954	0.003
hsa_circ_0002387	TNIK	333	1.809	3.505	0.021
chr5:162909647-162911251:+	HMMR	577	1.657	3.154	0.021
hsa_circ_0072760	CCNB1	159	1.642	3.122	0.018
hsa_circ_0046702	YES1	279	1.481	2.791	0.016
hsa_circ_0030991	CUL4A	187	1.349	2.548	0.015
hsa_circ_0004976	ASXL2	372	1.337	2.526	0.021
hsa_circ_0007161	YAF2	274	1.262	2.399	0.041
hsa_circ_0044623	LUC7L3	220	1.250	2.378	0.004
**Down-regulated**					
hsa_circ_0009024	TXLNG2P	298	−4.336	0.050	0.000
hsa_circ_0008297	DDX3Y	434	−3.597	0.083	0.000
hsa_circ_0001953	ZFY	662	−2.500	0.177	0.005
chr5:178043882-178044435:-	CLK4	158	−1.990	0.252	0.001
hsa_circ_0006660	CHPT1	568	−1.895	0.269	0.000
chr19:11759172-11759299:+	ZNF833P	127	−1.664	0.316	0.020
hsa_circ_0003068	SYNE1	290	−1.660	0.316	0.025
chr11:66372959-66373063:+	CCS	104	−1.642	0.321	0.026
hsa_circ_0028904	RNF10	137	−1.634	0.322	0.002
chr1:41474465-41474562:+	CTPS1	97	−1.575	0.336	0.018

### Differentially expressed lncRNAs (DElncRNAs) between T2DM and DD

**Figure 4:**
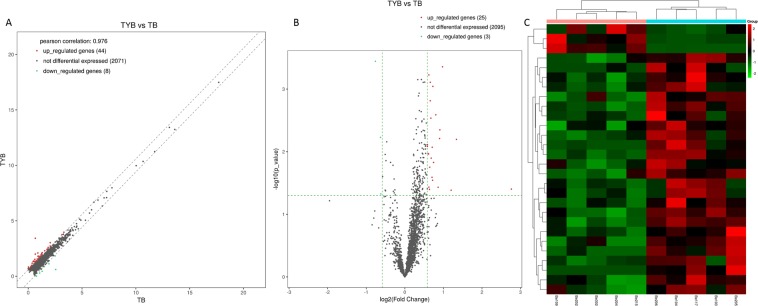
Plots of lncRNA. Scatter plot (**A**), volcano-Plot (**B**) and hierarchical clustering (**C**). X-axis and y-axis represent FPKM mean values (log2 transitions) of two groups. The vertical lines correspond to a 2-fold change in expression (up or down), and the horizontal line represents p = 0.05. The red point in the plot represents the DElncRNAs with statistical significance.

Compared with T2DM group, there were 25 up-regulated lncRNAs and 3 down-regulated lncRNAs detected in DD group (Fold Change > 1.5 and P < 0.05, Fig. 4, Supplementary data 3). XIST and AP000350.5 were up-regulated and down-regulated expressions of the most significant lncRNAs, respectively, which were 6.7 and 0.59 fold change higher than those in the T2DM group, respectively (Table 4). We found that XIST belongs to intergenic lncRNA and is closely related to the pathogenesis of T2DM with depression. We will use it as an important research target for the development of DD. The expression levels of three selected lncRNAs (XIST, RP11-706O15.3, and RP11-415F23.2), as determined by quantitative real-time PCR, were consistent with the sequencing results (Fig. 2), verifying the accuracy and reliability of the m sequencing data.

**Table 4:** Top 10 up-regulated lncRNAs and down-regulated lncRNAs.

Track id	Gene Name	log2FC	Fold Change	P值	q值
**Up-regulated**					
ENSG00000229807.10_2	XIST	2.763	6.787	0.040	0.610
ENSG00000234449.2_1	RP11-706O15.3	1.337	2.527	0.006	0.610
ENSG00000205663.5_1	RP11-706O15.5	1.201	2.298	0.041	0.610
ENSG00000271964.1_2	RP11-415F23.2	0.977	1.969	0.000	0.496
ENSG00000279995.1_2	RP11-1250I15.1	0.909	1.877	0.004	0.610
ENSG00000271109.1_1	CTC-523E23.11	0.902	1.868	0.006	0.610
ENSG00000205662.2_1	RP11-706O15.7	0.863	1.819	0.037	0.610
ENSG00000244620.1_1	AL122127.25	0.803	1.744	0.003	0.610
ENSG00000204282.4_2	TNRC6C-AS1	0.746	1.677	0.015	0.610
ENSG00000225938.1_1	RP4-575N6.4	0.727	1.655	0.001	0.511
**Down-regulated**					
ENSG00000273295.1_1	AP000350.5	−0.762	0.590	0.000	0.496
ENSG00000218537.1_1	MIF-AS1	−0.631	0.646	0.006	0.610
ENSG00000271869.1_1	RP11-51J9.5	−0.622	0.650	0.047	0.610

### Classification of differentially expressed lncRNAs

**Figure 5:**
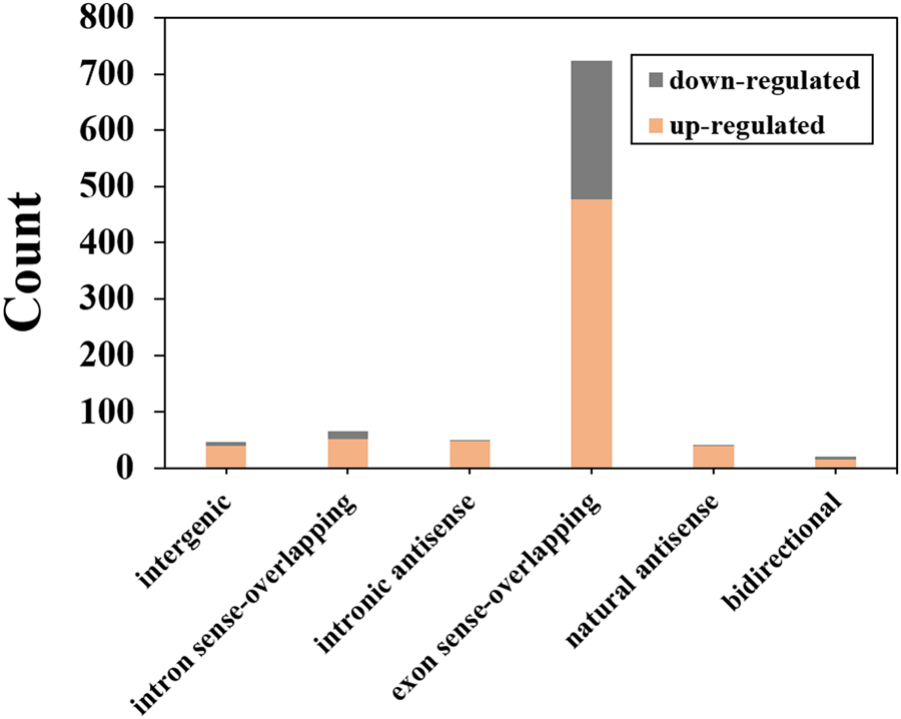
Classification of differentially expressed lncRNAs.

DElncRNAs are divided into six classes, including exon sense-overlapping, intron sense-overlapping, intergenic, natural antisense, intronic antisense and bidirectional, based on the relative positions of the DElncRNAs and their protein-coding genes on the genome (Fig. 5). In the two groups of DElncRNAs, the number of lncRNAs of the exon sense-overlapping type was the most, there were 477 up-regulated and 247 down-regulated expressions; followed by intron sense-overlapping lncRNAs, a total of 66 DElncRNAs were belonged to this type, 52 of them are up-regulated and 14 is down-regulated. There are 50 and 42 lncRNA belong to intronic and natural antisense, respectively. In summary, we found that the DElncRNAs in the DD group were mainly located in the exon sense-overlapping part, followed by intron sense-overlapping part.

### Co-analysis of differentially expressed antisense lncRNAs and mRNAs

Antisense lncRNAs can induce epigenetic changes in chromatin and DNA, thereby affecting the corresponding sense mRNA expression^14^. Therefore, we integrated the antisense lncRNAs and their sense mRNAs to further infer the function of differential lncRNAs. We found that in DD patients, one antisense lncRNA may have multiple sense mRNAs corresponding to it. In addition, there are two types antisense (intronic and natural antisense) of the relationship between antisense lncRNAs and sense mRNA. In this study, we found a total of 34 sense mRNAs corresponding to antisense lncRNAs, of which 20 were intronic antisense and 14 were natural antisense (Table 5).

**Table 5:** Analysis of differential antisense lncRNAs and associated sense mRNAs.

lncRNA id	log2FC	P	Relationship	mRNA id	log2FC	p
**Up-regulated**						
ENST00000474863	0.935	0.049	natural antisense	ENST00000340934.9_1	0.184	0.655
ENST00000474863	0.935	0.049	natural antisense	ENST00000612948.4_1	−0.111	0.838
ENST00000532943	0.774	0.021	intronic antisense	ENST00000531974.5_1	0.047	0.832
ENST00000484377	0.754	0.021	natural antisense	ENST00000290219.10_1	−0.197	0.454
ENST00000484377	0.754	0.021	natural antisense	ENST00000381995.5_1	0.468	0.613
ENST00000484377	0.754	0.021	natural antisense	ENST00000405436.5_2	−1.392	0.077
ENST00000613908	0.747	0.012	intronic antisense	ENST00000311052.9_2	0.918	0.099
ENST00000613908	0.747	0.012	intronic antisense	ENST00000396593.6_2	−0.678	0.169
ENST00000613908	0.747	0.012	intronic antisense	ENST00000562843.5_1	0.682	0.045
ENST00000613908	0.747	0.012	intronic antisense	ENST00000567908.5_1	0.010	0.981
ENST00000613908	0.747	0.012	intronic antisense	ENST00000618335.4_2	0.341	0.365
ENST00000613908	0.747	0.012	intronic antisense	ENST00000619881.4_2	0.469	0.321
ENST00000497437	0.730	0.031	natural antisense	ENST00000372517.6_2	0.177	0.575
ENST00000606713	0.719	0.029	intronic antisense	ENST00000334133.8_1	0.607	0.160
ENST00000448942	0.714	0.000	intronic antisense	ENST00000237289.8_2	0.002	0.991
ENST00000448942	0.714	0.000	intronic antisense	ENST00000420009.5_2	−0.061	0.835
ENST00000448942	0.714	0.000	natural antisense	ENST00000433680.1_2	−0.219	0.517
ENST00000448942	0.714	0.000	intronic antisense	ENST00000612899.4_2	−0.072	0.677
ENST00000462700	0.702	0.014	natural antisense	ENST00000335351.7_1	0.175	0.378
ENST00000554945	0.694	0.001	intronic antisense	ENST00000556994.5_1	0.195	0.740
ENST00000484282	0.692	0.047	natural antisense	ENST00000513973.5_1	0.155	0.173
ENST00000441423	0.685	0.012	intronic antisense	ENST00000538264.1	0.416	0.050
ENST00000608684	0.676	0.003	intronic antisense	ENST00000426517.1_1	−0.339	0.509
ENST00000541574	0.646	0.018	natural antisense	ENST00000377071.8_1	0.129	0.718
ENST00000473954	0.638	0.009	intronic antisense	ENST00000259873.4_1	0.147	0.482
ENST00000565600	0.623	0.001	intronic antisense	ENST00000329410.3_1	−0.028	0.868
ENST00000423354	0.603	0.011	natural antisense	ENST00000405006.8_2	0.054	0.879
ENST00000423354	0.603	0.011	natural antisense	ENST00000405975.6_2	0.053	0.915
ENST00000503571	0.594	0.016	intronic antisense	ENST00000362003.5	0.792	0.177
ENST00000503571	0.594	0.016	intronic antisense	ENST00000470161.2	0.577	0.144
ENST00000503571	0.594	0.016	intronic antisense	ENST00000505655.2	−0.779	0.106
ENST00000503571	0.594	0.016	intronic antisense	ENST00000521023.2	0.197	0.235
**Down-regulated**						
ENST00000406213	−0.631	0.006	natural antisense	ENST00000215754.7_1	0.929	0.046
ENST00000532446	−1.594	0.044	natural antisense	ENST00000307980.6_1	0.094	0.752

### GO analysis

**Figure 6:**
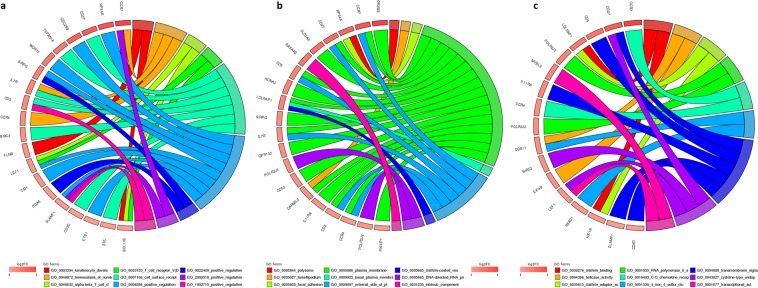
Circle drawing represented the mainly involved biological processes (**a**), cellular component (**b**) and molecular function (**c**).

GO analysis found that DEmRNAs in the blood of DD patients were mainly enriched in 20 BP entries, 22 CC entries, and 13 MF entries, respectively. In the BP category, DEmRNAs are mainly enriched in homeostasis of number of cells、cell surface receptor signaling pathway and hypothalamus development; in CC entries, DEmRNAs are mainly enriched in the external side of plasma membrane、 hemidesmosome and neuronal cell body, etc. In addition, DEmRNAs are mainly concentrated in the cysteine-type endopeptidase inhibitor activity involved in apoptotic process、RNA polymerase II activity and C-C chemokine receptor activity in the MF entry. Therefore, we speculate that the occurrence and development of DD may be related to GO items such as brain and neuropsychiatric system development and chemokines (Fig. 6).

### KEGG Pathway analysis

**Figure 7:**
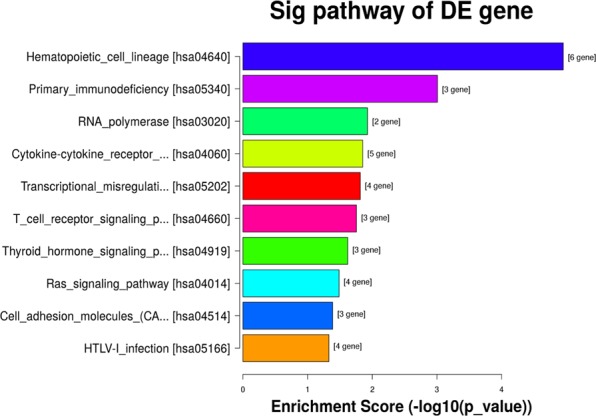
KEGG pathway analysis. Sorts from low to high according to P-values and the abscissa indicates enrichment score (−log10 (P value)).

Pathway analysis showed that DEmRNAs were involved in 20 pathways. Among them, there were 11 statistically significant pathways (P < 0.05): Hematopoietic cell lineage, RNA polymerase, Cytokine-cytokine receptor interaction, T cell receptor signaling pathway, Thyroid hormone signaling pathway, ras signaling pathway, Cell adhesion molecules, CAMs, HTLV-I infection and Ribosome (Fig. 7). The T cell receptor and Ribosome signaling pathway are closely related to the occurrence and development of DD. A total of three DEmRNAs participate in the T cell receptor pathway and five DEmRNAs participate in the C-C chemokine receptor activity pathway. CD27 is an important target we screened and it is involved in Cytokine-cytokine receptor interaction. Therefore, we speculate that Cytokine-cytokine receptor interaction plays an important role in the development of DD.

### Differentially lncRNAs Gene Set Enrichment Analysis (GSEA Prerank)

**Figure 8:**
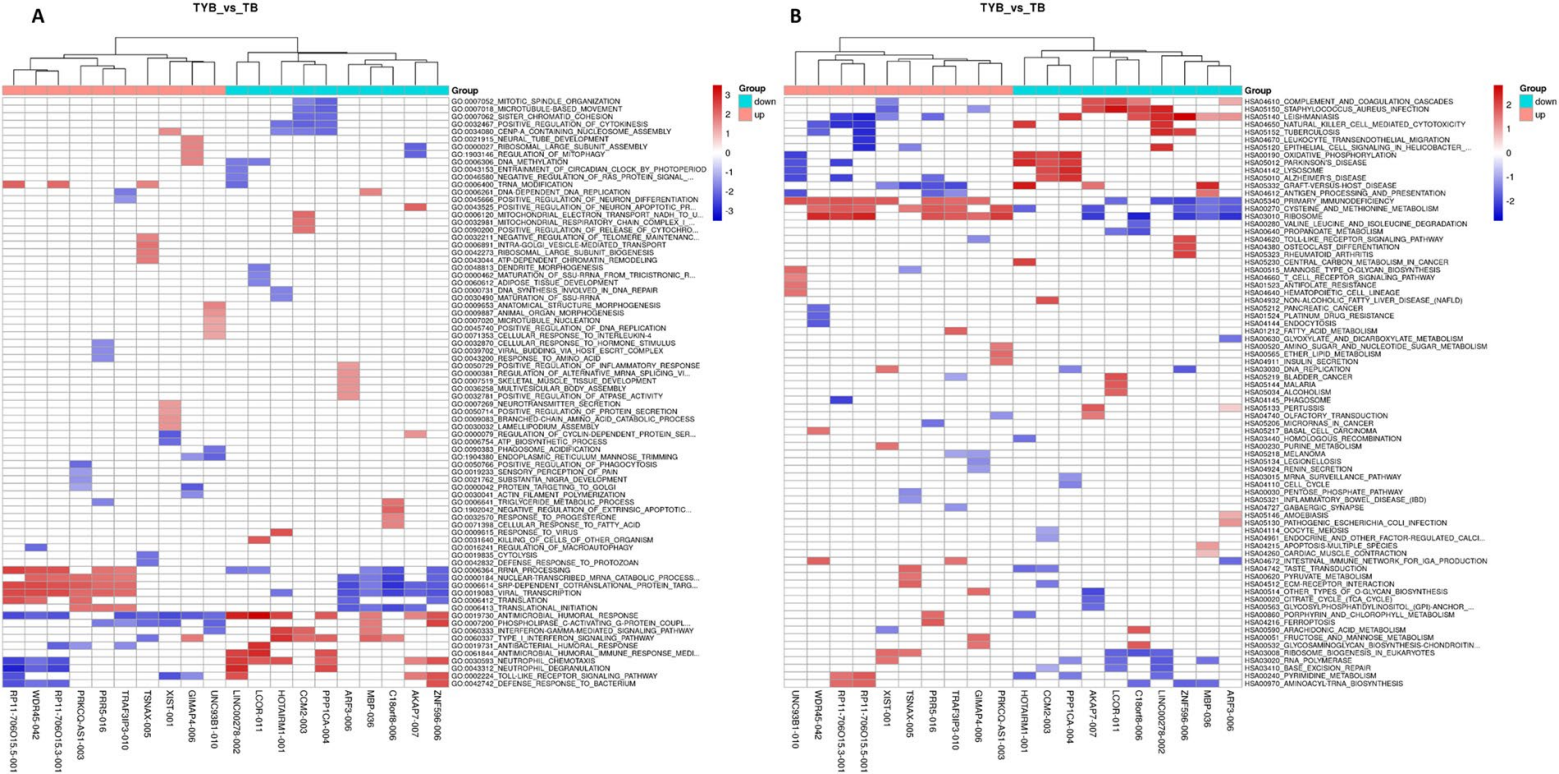
Difference lncRNAs GSEA Prerank. (**A**) Biological Process and (**B**) KEGG Pathway, each row represents a functional entry, and each column represents an lncRNA.

As shown in the Fig. 8, differential lncRNAs with the same biological process and the same pathway are clustered together. Compared with the T2DM group, we found that the DElncRNAs in the DD group were mainly related to mitotic spindle organization, microtubule based movement, sister chromatid, positive regulation of cytokinesis and other BP and natural killer cell mediated cytotoxicity, leukocytes transendothelial migration, oxidative phosphorylation, T cell receptor signaling pathway, cysteine, methionine metabolism and other pathways. GSEA analysis makes up for the insufficiency of single lncRNAs in the analysis, providing insights for the next in-depth study of the role of differential lncRNAs in the development of DD.

### CNC network analysis

**Figure 9:**
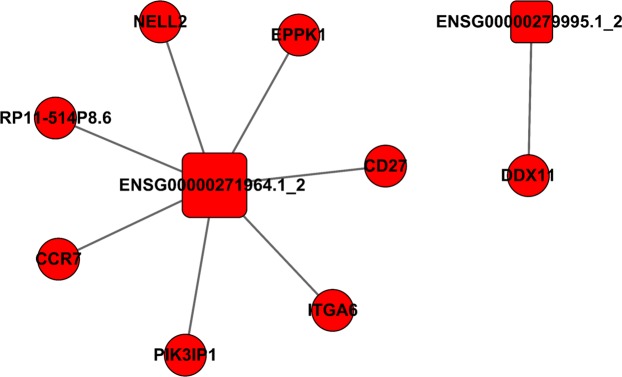
CNC network. Squares and circles represent lncRNA and mRNA, respectively. The solid line is positively correlated.

We selected two significantly up-regulated lncRNAs (ENSG00000271964.1_2 and ENSG00000279995.1_2) to construct the lncRNA-mRNA interaction network using Cytoscape v2.8.3 (Fig. 9). According to the network, we found a total of 8 mRNAs related to these two differentially expressed lncRNAs. Specifically, ENSG00000271964.1_2 was associated with seven mRNAs, including the important differential mRNA-CD27, CCR7, NELL2, and EPPK1 that we screened; whereas ENSG00000279995.1_2 was positively correlated with DDX11. These DEmRNA and lncRNA will be the focus of our further research.

## Discussion

By annotating the DElncRNAs, we found that up-regulated expression of lncRNA-XIST was associated with the development of DD. Genetic studies have found that inactive X chromosomes often lead to psychiatric symptoms in patients^15^. XIST (X inactive specific transcript) is a unique length of 19 kb lncRNA, which is the major lncRNA regulating X chromosome inactivation (XCI)^16,17^. XIST was significantly overexpressed in lymphoblasts of patients with severe depression, which may be due to compensatory responses caused by XCI deficiency^18^. The unique pattern of neuronal expression of the XIST chromosomal gene may lead to the prevalence of certain neuropsychiatric disorders. Our study found that compared with the T2DM group, the expression level of XIST in the DD group was significantly upregulated. This result is consistent with previous reports, indicating that the expression level of lncRNA-XIST is indeed correlated with the onset of depression and is expected to be useful as a biomarker for diagnosing mental disorders in patients. However, the specific relationship between them will be our future research direction.

In addition, up-regulated expression of RP11-706O15.3 and RP11-415F23.2 also attracted our attention, with an up-regulation multiple of 2.527 and 1.969, respectively. Although these two lncRNAs were not found in previous studies, due to their significant differential expression, we suspect that they may play an important role in the development of DD.

To gain insight into the regulatory functions of lncRNAs, we focused on differentially expressed antisense lncRNAs. Antisense lncRNAs are RNA molecules that are transcribed from the antisense strand and regulate their corresponding sense mRNAs at the transcriptional and post-transcriptional levels^19–21^. In this study, we found a total of 34 target genes associated with antisense lncRNAs. Among them, interferon γ receptor 2 (IFNGR2) corresponds to antisense lncRNA- ENST00000484377. Interferon γ is a pleiotropic protein secreted by immune cells, and its signal exerts an immune action through interferon γ receptors^22^. The gene corresponding to antisense lncRNA-ENST00000613908 is calcium-regulated heat-stable protein 1 (carhsp1), a biomarker of diabetic complications, which can inhibit the hepatic gluconeogenesis gene expression by inhibiting the expression level of PPARα^23^. Therefore, carhsp1 can be used as a molecular target for diagnosis and treatment of diabetic complications. In addition, we found that up-regulated expression of antisense lncRNA-ENST00000448942 in the DD group could down-regulate TNFAIP3 expression. Studies have shown that the expression level of mRNA-TNFAIP3 is inversely related to the degree of depression^24^. Therefore, we can use up-regulated antisense lncRNA-ENST00000448942 as a molecular target for diagnosing DD.

The function and regulation mechanism of most lncRNAs remains unclear. Therefore, we constructed mRNA expression profiles to further screen DEgenes associated with DD. Inflammation is considered to be an important pathogenesis of neuropsychiatric diseases, and inflammatory immune activation is closely related to the development of depression^25^. Chemokines are part of the immune system and have an impact on brain cognition and learning behaviors, including anxiety^26^. The chemokine receptor CCR7 (CC-chemokine receptor 7) is a GTP-protein-coupled transmembrane receptor for CC chemokines and is normally expressed in the cell membranes of immune cells and endothelial cells. The study found that the activation of the pro-inflammatory surface marker CCR7 is associated with depressive behavior, and that activation of CCR7 may aggravate anxiety and depression behavior in the male mice^27,28^. Compared with the T2DM group, the expression level of CCR7 in the DD group was significantly upregulated (Fold change = 2.05), which may be related to the occurrence of depression.

In our study, some immune-related DEmRNAs were also found. B cells play a key role in the inflammatory response by secreting proinflammatory and anti-inflammatory factors. It has been found that the up-regulated expression of CD27, a molecule on the surface of B cells, is associated with severe depression^13^. In our study, the expression level of CD27 was significantly higher in the DD group than in the T2DM group (Fold change = 1.89). This suggests that the occurrence of DD may be related to the activation of CD27, and they may be used as targets for preventing and improving DD in future studies.

We analyzed the function of DEmRNAs through the GO and KEGG pathways and found that they are involved in many aspects of the progression of T2DM and involve a number of related pathways of depression such as CC chemokine receptor activity, cytokine-cytokine receptor interactions and T cell receptor signaling pathways. Among them, the “T cell receptor signaling pathways” and the “CC chemokine receptor activity^29^” have an important correlation with the occurrence and development of DD. Therefore, we speculate that the occurrence and development of DD may be related to GO items and pathways such as brain, neuropsychiatric system development, and chemokines.

Depression is a complex disease that affects certain specific brain circuits and components of the nervous system and is induced by the interrelationship of epigenetics and the environment^30^. CircRNA-TFRC (transferrin receptor, TFRC) is associated with insulin resistance, and the overexpression of TFRC can aggravate the risk of type 2 diabetes and metabolic diseases^31,32^. Our study found that the expression level of TFRC in blood samples of DD patients was significantly higher than that of T2DM patients, indicating that DD patients had more severe insulin resistance and symptoms of diabetes than T2DM. Our results provide evidence from the molecular level that T2DM with depression is more dangerous than T2DM alone. In addition, another study found that TFRC expression levels are associated with mental disorders, suggesting that we can use TFRC as the molecular target for the study of the occurrence and development mechanism of DD^33^.

Another up-regulated circRNA-TNIK (Traf2- and Nck-interacting kinase, TNIK) is associated with depression-related mental disorders^34^. TNIK is a highly expressed serine/threonine kinase in the brain that regulates synaptic composition and activity density by stabilizing critical postsynaptic levels and has a favorable role in dendritic development and synaptic transmission^35^. In this study, compared with the T2DM group, we found that the expression level of TNIK in the DD group was significantly increased (Fold Change = 3.505, P < 0.05), suggesting that we can focus on the function of TNIK in the next study.

## Materials and Methods

### Sample information description

This study was conducted in accordance with the guidelines of the Declaration of Helsinki, and the study protocol was approved by the Ethical Committee of Beijing University of Chinese Medicine (BUCM) (2016BZHYLL0105). After fully explaining the purpose and nature of all the procedures used, consent has been obtained from each patient or subject and informed consent has been signed. Enrolled patients were divided into T2DM group (patient ID: 302, 109, 215, 202, and 204) and DD group (patient ID: 206, 103, 205, 117, and 104). DD patients were screened in T2DM patients using the SDS and the PHQ9.

### Library construction and Illumina sequencing

The total RNA was extracted according to our previous experimental method^36^. Briefly, using TRIzol reagent (Invitrogen, Carlsbad, CA, USA) and purified with an RNeasy Mini Kit (Qiagen, Hilden, Germany) according to manufacturer protocol. Enrich mRNA with oligo (dT) beads (rRNA removal kit for RNA degradation or prokaryotic); RNA sequencing library are completed by the KAPA Stranded RNA-Seq Library Prep Kit (Illumina). Finally, the constructed library was checked with the Agilent 2100 (NanoDrop ND-1000). The sequencing library was denatured to a single-stranded DNA by 0.1 M NaOH, diluted to a concentration of 8 pM, and then amplified *in situ* on the TruSeq SR Cluster Kit v3-cBot-HS (#GD-401-3001, Illumina), and the resulting fragment at the end was used the Illumina HiSeq 4000 sequencer was sequenced for 150 cycles.

### Quantitative analysis of DERNAs

Transcript abundance calculations were performed by software StringTie and expression unit expressed by FPKM (Fragments Per Kilobase of gene/transcript model per Million mapped fragments). Genes and lncRNA were expressed at a threshold of FPKM ≥ 0.5 and the average number of genes with FPKM exceeding 0.5 in each group was considered as expressed in the group and statistically analyzed. The expression of circRNA (Backsplice junction reads count) was quantified by the software STAR and then calculated by CIRCexplorer2 for Backsplice junction reads. CircRNA expression threshold is that CircRNAs with mean CPM exceeding 100 in each group are considered as expressed in the group and statistically analyzed.

### Principal component analysis

Principal Component Analysis (PCA) is a classification of data presented by the expression data of the sample. The visual distribution of the sample between the experimental group and the control group was obtained by PCA analysis. We used PCA analysis of genes with significant differences (P value ≤ 0.05) in mean values across (ANOVA) all samples and mapped PCA (using all genes for PCA plots).

### RT-PCR

Extraction total RNA from another 20 blood samples using Trizol reagent and reverse-transcribed into cDNA according to the manufacturer’s instructions. The reverse transcriptase reactions contained 1.5 µg RNA, 0.5 μg/μL random primers (N9, 1 μL), 2.5 mM dNTPs mix (1.6 µL), 5× first-strand buffer (4.0 μL), 0.1 M dithiothreitol (DTT, 1 µL), RNase inhibitor (0.3 µL), and Superscript III RT (0.2 µL). All data were normalized to beta-actin data to calculate the relative concentrations of mRNA and lncRNA. Details of the genes and primers are listed in Table 6.

**Table 6:** mRNA and lncRNA primers.

Gene name	Forward and Reversed primer	bp
**mRNA**		
β-actin (H)	F:5′ GTGGCCGAGGACTTTGATTG 3′ R :5′ CCTGTAACAACGCATCTCATATT 3′	73
CD27	F:5′ GGCACTGTAACTCTGGTCTTC 3′ R :5′ ACTGACATAAGGTAAGTGGGTG 3′	194
CCR7	F:5′ AGCAACTCAACATCGCCTACG 3′ R :5′ CAAGAAAGGGTTGACGCAGC 3′	71
**LncRN**		
XIST	F:5′ GATCCCATTGAAGATACCACG 3′ R:5′ CAACCCATCCAAGTAGATTAGC 3′	181
RP11-706O15.3	F:5′ GTCTAACTGATTCCATCGCCC 3′ R:5′ GACACATGATTCCAGGTGAGCT 3′	257
RP11-415F23.2	F:5′ AACACCCTCCACTGTAGACTGA 3′ R:5′ TTGGCTGTATTTGCTATGGG 3′	165
DLGAP1-AS1	F:5′ CTCAGGAACCTGGCAACAGTG 3′ R:5′ GCTGTGGTTACATCCGTGGAA 3′	165

### Bioinformatics analysis

We used topGO software and the newest KEGG database (Kyoto Encyclopedia of Genes and Genomes) for GO and Pathway analysis to obtain significant enriched GO terms and important biological functions involved in DEgenes, ultimately identifying and differentiating the most relevant pathway for DEgenes.

GSEA is a computational method used to determine whether a given gene set has significant differences among different groups^37^. Briefly speaking, we follow the Subramanian method^38^, using Java software, first calculate the Enrichment Score (ES), then estimate the importance of the ES, and finally evaluate their importance by adjusting multiple hypothesis tests.

### Coding-noncoding network (CNC) analysis

An lncRNA-mRNA regulatory network was constructed based on the inter-regulatory association between DEgenes in the blood between DD and T2DM patients using Cytoscape v2.8.2 software (http://www.cytoscape.org/).

### Statistical analysis

SPSS software (Version 20.0) was used in this study for the statistical analyses. The results are expressed as mean ± SEM (standard error). Comparisons between multiple groups was analyzed using one-way ANOVA analysis. Two-sided P values < 0.05 were considered statistically significant.

## Conclusions

The onset of DD is a complex and highly regulated biological process. In this experiment, we compared the lncRNA, circRNA and mRNA between DD and T2DM and finally screened out the DEgenes profile of DD. Through the annotation of DEgenes, function, some molecules closely related to the development of DD were discovered, such as TNIK, TFRC, XIST, RP11-706O15.3, RP11-415F23.2, CD27 and CCR7. We expect that these DERNA molecules will become attractive biomarkers to enhance the research and diagnostic potential associated with DD.

## Supplementary information

Supplement table 1

Supplementary data 1

Supplementary data 2

Supplementary data 3

## Data Availability

The datasets used and analyzed during the current study are available from the first or corresponding authors on reasonable request.
