# Supplementary material for: Relationships of Non-coding RNA with diabetes and depression
Source: Sci Rep. 2019 Jul 24;9:10707. doi: 10.1038/s41598-019-47077-9 (PMC6656886; doi:10.1038/s41598-019-47077-9)
Supplement: Supplementary file 1 — Supplement table 1 [file 41598_2019_47077_MOESM1_ESM.doc]

**Relationships of Non-coding RNA with diabetes and depression**

Tian An^1,#^, Jing Zhang^2,#^, Yue Ma^1^, Juan Lian ^1^, Yan-Xiang Wu^1^, Bo-Han Lv^1^, Meng-Hua Ma^2^, Jun-Hua Meng^2^, Yun-Tao Zhou^2^, Zhi-Yong Zhang^2^, Qing Liu^3^, Si-Hua Gao^1^*, Guang-Jian Jiang^1^*

**Supplementary Table 1. Clinical characteristics of the participants.**

| **ID** | **Group** | **Age** | **Gender** | **Total coarse points** | **Standard score** | **PHQ-9** |
| --- | --- | --- | --- | --- | --- | --- |
| **302** | T2DM | 62 | Female | 37 | 46 | 1 |
| **109** | T2DM | 70 | Female | 32 | 40 | 4 |
| **215** | T2DM | 41 | Female | 25 | 31 | 2 |
| **202** | T2DM | 61 | male | 33 | 41 | 3 |
| **204** | T2DM | 65 | Female | 31 | 38 | 4 |
| **206** | DD | 60 | Female | 49 | 61 | 17 |
| **103** | DD | 48 | Female | 46 | 57 | 6 |
| **205** | DD | 62 | male | 44 | 55 | 5 |
| **117** | DD | 55 | Female | 49 | 61 | 12 |
| **104** | DD | 54 | Female | 40 | 50 | 6 |
